# Development of PSP1, a Biostimulant Based on the Elicitor AsES for Disease Management in Monocot and Dicot Crops

**DOI:** 10.3389/fpls.2018.00844

**Published:** 2018-07-24

**Authors:** Nadia R. Chalfoun, Sandra B. Durman, Florencia Budeguer, María d. P. Caro, Romina P. Bertani, Pía Di Peto, Sebastián A. Stenglein, María P. Filippone, Enrique R. Moretti, Juan C. Díaz Ricci, Björn Welin, Atilio P. Castagnaro

**Affiliations:** ^1^Instituto de Tecnología Agroindustrial del Noroeste Argentino – Consejo Nacional de Investigaciones Científicas y Técnicas–Estación Experimental Agroindustrial Obispo Colombres, Las Talitas, Argentina; ^2^Bayer S.A., Argentina – Crop Science LATAM 2, Crop Science Research, Buenos Aires, Argentina; ^3^Instituto Superior de Investigaciones Biológicas – Consejo Nacional de Investigaciones Científicas y Técnicas and Instituto de Química Biológica “Dr. Bernabé Bloj”, Facultad de Bioquímica, Química y Farmacia, Universidad Nacional de Tucumán, San Miguel de Tucumán, Argentina; ^4^Laboratorio de Biología Funcional y Biotecnología, Universidad Nacional del Centro de la Provincia de Buenos Aires-Comisión de Investigaciones Científicas de la Provincia de Buenos Aires and Instituto de Investigaciones en Biodiversidad y Biotecnología – Consejo Nacional de Investigaciones Científicas y Técnicas, Azul, Argentina; ^5^ANNUIT S.A., Buenos Aires, Argentina

**Keywords:** induced resistance, plant defense, strawberry anthracnose, soybean target spot, sugarcane red stripe, wheat Fusarium head blight

## Abstract

In this work, we present a novel biostimulant for sustainable crop disease management, PSP1, based on the plant defense-elicitor AsES, an extracellular protease produced by the strawberry fungal pathogen *Acremonium strictum*. Fungal fermentation conditions and downstream processing were determined to maximize extracellular protein production, product stability and a high plant defense-eliciting activity, as monitored by anthracnose resistance in supernatant-treated strawberry plants subsequently infected with a virulent strain of *Colletotrichum acutatum*. Fermentation batches were shown to reduce anthracnose development by 30–60% as compared to infected non-treated plants. Product formulation was shown to be stable for 6 months when stored at temperatures up to 45°C and toxicological tests showed that PSP1 was harmless to beneficial organisms and non-toxic to mammalian species at concentrations 50 times higher than those used in plant experiments. Furthermore, disease protection studies using dilutions of PSP1 indicated that there is a minimum threshold protease activity needed to induce pathogen defense in strawberry and that this induction effect is dose-independent. A significant characteristic of PSP1 is its broad-range protection against different diseases in various crop species. In soybean, PSP1 reduced the symptomatology by 70% of *Corynespora cassiicola*, etiological agent of the target spot. This protection effect was similar to the commercial inducer BION 500 WG based on BTH, and both products were shown to induce an oxidative burst and up-regulated *PR1*-gene expression in soybean. Furthermore, a double PSP1-treatment on greenhouse-grown sugarcane plants provided protection against bacterial red stripe disease caused by *Acidovorax avenae* and a double foliar application of PSP1 on field-grown wheat plants significantly increased resistance against *Fusarium graminearum*, causal agent of head blight disease, manifested mainly in an increased seed germination rate. In summary, these disease protection studies demonstrated an effective control against both bacterial and fungal pathogens in both monocot and dicot crop species, which together with its low production cost, effectiveness at low concentrations, long shelf-life, tolerance to high temperatures, harmlessness to non-target organisms and simple handling and application, make PSP1 a very promising candidate for effective and sustainable disease management in many crop species.

## Introduction

Diseases caused by fungal, oomycete, bacterial, and viral pathogens produce severe reductions in yield and quality of crop harvests, which in many cases lead to important economic losses for farmers (Vidhyasekaran, 2004). Therefore, to combat pathogens and reduce infestations, farmers apply a multitude of different pesticides that in many cases have a negative effect on the environment and human/animal health. Moreover, as a result of the extensive usage of chemicals in disease crop management, development of resistance to modern fungicides in field populations of fungal and oomycete pathogens have increased (Fernández-Ortuño et al., 2015; Saville et al., 2015).

Due to the described problems much effort is concentrated on finding new efficient, economical, low environmental-impact options to control pathogens. One of the most promising approaches is the use of plant defense elicitors, alone or in combination with pesticides or beneficial microorganisms (Walters et al., 2013). Elicitors or plant defense activators normally induce an incomplete, broad range, systemic resistance (Walters and Fountaine, 2009) as displayed by a significant reduction in disease symptoms caused by different types of pathogens (Kuć, 2006).

Over the last two decades, several resistance elicitors have been described, and they have been classified into two main groups, molecules that are, or mimic, phytohormones or compounds that resemble the presence of a plant enemy (Heil, 2014). Therefore, pathogen protection in plants can be induced by the exogenous application of phytohormones that activate systemic resistance responses such as salicylic acid (SA) and jasmonic acid (JA) (Pieterse et al., 2009). In addition, some volatile derivatives or artificial compounds that structurally resemble these hormones and cause similar plant responses have been characterized, such as 2,6-dichloroisonicotinic acid (INA) or benzothiadiazole (BTH), also known as acibenzolar-*S*-methyl (ASM). In the second group, resistance can be induced by the application of: (i) molecules that indicate the presence of pathogens or microbes and thus are perceived by plants as ‘pathogen-associated molecular patterns’ (PAMPs) or ‘microbe associated molecular patterns’ (MAMPs) and of (ii) plant extracts containing break-down products known as ‘damage-associated molecular patterns’ (DAMPs). PAMPs- or MAMPs-like molecules can form part of the cell wall of microorganisms, be commonly secreted by them or represent other invariable characteristics of microbial surfaces (Heil, 2014). In addition to the two aforementioned groups of elicitors, a third type of compounds including mineral nutrients such as phosphorus (P), potassium (K), and silicon (Si), fertilizers containing phosphite (phosphorous acid) and the non-protein amino acid DL-3-amino-*n*-butanoic acid (BABA) have been shown to induce pathogen defense responses and enhance plant disease protection (Reglinski et al., 2014).

Many studies have demonstrated the successful use of different elicitors for suppression of diseases in a wide variety of crops (Lyon et al., 1995; Aziz et al., 2006; Elmer and Reglinski, 2006; Renard-Merlier et al., 2007; French-Monar et al., 2010). The first disease resistance activator, probenazole (3-allyloxy-1,2-benzisothiazole-1,1-dioxide), was registered in Japan more than 30 years ago as Oryzemate to control rice blast (Iwata et al., 2004). Since then, many other chemical and biological activators have been commercially developed and registered as agricultural products including BION or Actigard (formulated with BTH; Syngenta, Switzerland), Milsana (*Reynoutria sachalinensis* extract; KHH BioScience, United States), Elexa (chitosan; SafeScience, United States; and Glycogenesys Inc., United States) and Messenger or ProAct (harpin protein; Plant Health Care, United States) also commercialized as Employ (H&T Health Promoter, United States) (Walters et al., 2013). Currently, commercial formulations based on different PAMP/MAMP compounds such as flagellin, elicitins, harpins, cerebrosides, rhamnolipids, Sm1 protein and yeast derived-elicitors, are being developed (Elmer and Reglinski, 2006; Iriti et al., 2011; Dafermos et al., 2012).

Switching on the plant innate immunity response through treatment with different PAMP/MAMP molecules has demonstrated the effectiveness of this technology to control diseases caused by virus, bacteria, oomycetes and a wide range of biotrophic, hemibiotrophic, and necrotrophic fungi in many different plant species (Iriti et al., 2011; Li et al., 2011; Choi et al., 2012; Dafermos et al., 2012; Sanchez et al., 2012; Quang et al., 2015). The use of induced resistance to improve plant protection against pathogens has mostly been implemented in intensive and greenhouse grown crops such as fruits, vegetables, and ornamentals, although there are a considerable number of examples of successful application in extensive crop production systems including maize, wheat, and barley (Vallad and Goodman, 2004; Reglinski et al., 2014). In addition to trigger protection against plant pathogens, some elicitors have also been shown to affect physiological responses in plants, resulting in an increased crop yield and quality through improved nutrient assimilation or increased tolerance to low or high temperatures and water abiotic stress (Khan et al., 2009; Calvo et al., 2014; Chuang et al., 2014).

Traditionally, defense elicitor-based products has been categorized as plant strengtheners or biopesticides but has recently been included in the more broad term biostimulant, defined as “a formulated product of biological origin that improves plant productivity as a consequence of the novel or emergent properties of the complex of constituents and not as a sole consequence of the presence of known essential plant nutrients, plant growth regulators, or plant protective compounds” (Yakhin et al., 2017). The main reason behind this new definition is to stimulate the development of harmless products of natural origin in agriculture production and facilitate regulation and registration governing these compounds (biostimulants).

We have previously described the isolation and characterization of AsES, an extracellular subtilisin-like protease produced by the opportunistic strawberry pathogen *Acremonium strictum* strain SS71 (Castagnaro et al., 2012; Chalfoun et al., 2013). This 34-kDa fungal protein has previously been shown to induce an innate defense response and generate protection against anthracnose in strawberry (Chalfoun et al., 2013; Hael-Conrad et al., 2018) and gray mold in Arabidopsis under controlled growing conditions (Hael-Conrad et al., 2015).

The important disease protection induced by AsES demonstrated in two different plant species and against different pathogens stimulated us to try and develop a novel broad-range crop disease control product, PSP1 (acronym for Plant Stimulation and Protection). In this study, we show the technological development of PSP1 from initial production and subsequent formulation, including tests of stability, quality control development, toxicological studies, adjustments of optimal concentration and application timing for disease control and testing of broad-range protection against diseases in three economically important crop species in Argentina, target spot caused by the fungus *Corynespora cassiicola* (Berk and M. A. Curtis) C. T. Wei in soybean (Ploper, 2011), red stripe (*Acidovorax avenae*) in sugarcane (Fontana et al., 2013), and head blight (*Fusarium* *graminearum* Schwabe) in wheat (Martinez et al., 2014).

## Materials and Methods

### Fermentation Conditions for SS71 *A. strictum* Growth

*Acremonium strictum* W. Gams strain SS71 (DSMZ accession number DSM 24396) was cultured in soybean meal broth (SMB) containing 0.5% soybean meal, 0.05% KH_2_PO_4_, 0.05% K_2_HPO_4_, 0.02% MgSO_4_, 0.002% CaCl_2_ and 0.002% of a microelement solution (1.2% FeSO_4_.7H_2_O, 0.25% MnSO_4_.H_2_O, 0.025% CoCl_2_.6H_2_O, 0.25% ZnSO_4_.7H_2_O, 0.05% CuSO_4_.5H_2_O, 0.02% Na_2_MoO_4_.2H_2_O, 0.5% citric acid) supplemented with 1% (w/v) glucose or in potato dextrose broth (PDB) with 2% (w/v) glucose as carbon source.

After pH-adjustment to 6.5, 50 ml-aliquots of each broth were autoclaved for 15 min and once cooled down to room temperature, antibiotics were added (streptomycin 100 mg/l and chloramphenicol 50 mg/l). Sterile broths were inoculated adding a conidia aqueous suspension, prepared from fungal colonies grown on Potato Dextrose Agar (PDA) plates, until reaching a concentration of 1 × 10^6^ conidia/ml of broth. Flasks containing PDB were incubated without agitation under continuous fluorescent light at 28°C while SMB flasks were incubated in a shaker (150 rpm) at 28°C until all glucose had been consumed.

Culture samples were taken periodically for microscopic observations and glucose consumption was assessed by the dinitrosalicylic acid (DNS) method that involves the oxidation of the aldehyde functional group (Miller, 1959). Fungal cultures, both in PDB and SMB, were harvested by centrifugation (13000 × *g*) at 4°C and collected supernatants were passed through a 0.22 μm pore filter. In the case of supernatants from SMB cultures, pH was thereafter adjusted to 5.5. Filter-sterilized supernatants were kept at 4°C until further use. Total soluble protein (TSP) content was determined using the Bradford colorimetric assay with bovine serum albumin (BSA) as protein standard (Bradford, 1976).

### Scaling Up of SS71 Fermentation

A 1.5 L fungal culture of *A*. *strictum* SS71 was obtained by fermentation in SMB medium in an air bubbling 4-L reactor during 3 days at 28°C and harvested by centrifugation at 9000 × *g* for 15 min to separate fungal biomass. Collected clarified supernatant was adjusted to pH 5.5, and sterilized by filtration trough 0.45 μm and 0.22 μm pore filters. All processed supernatants (named PSP1) were kept at 4°C until further use. To study thermal stability, 250 ml-aliquots of processed supernatants were kept at 37°C or 45°C for 6 months and thereafter tested for their capability to induce strawberry pathogen defense and residual protease activity (both procedures described below). TSP content was determined for different production batches using the Bradford colorimetric assay as indicated above.

### Purification of the Protease AsES

AsES protein was purified from the supernatant of an *A*. *strictum* SS71 culture as previously described (Chalfoun et al., 2013). In summary, the procedure included a membrane ultrafiltration of the concentrated supernatant (30-kDa cut-off filter pore), followed by two steps of fast protein liquid chromatography (FPLC) separation, first by anionic exchange (Q-Sepharose, pH 7.5) and secondly by hydrophobic interaction (phenyl-Sepharose). Purity grade was analyzed by 2D-PAGE and C18-HPLC as described previously (Chalfoun et al., 2013) and pure protein was lyophilized and kept at 4°C until use.

### Measurement of the Subtilisin-Like Proteolytic Activity

Proteolytic activity in PSP1 production batches was determined by enzymatic hydrolysis of the chromogenic peptide *N*-Succinyl-Ala-Ala-Pro-Phe-*p*-nitroanilide (Suc-AAPF-*p*NA; Sigma) (Chalfoun et al., 2013). Briefly, 10 μl of PSP1 product was diluted in 20 mM Tris–HCl (pH 7.5) to a final volume of 500 μl. After 2 min of preincubation of the reaction mixture without substrate at 37°C, 10 μl of 5 mM Suc-AAPF-*p*NA (final concentration of 0.1 mM) was added and the mixture incubated for 30 min under the same conditions. Reactions were stopped by addition of phenylmethylsulfonyl fluoride (PMSF) to a final concentration of 1 mM and the enzymatic activity was quantitatively assayed according to Moallaei et al. (2006). Lyophilized AsES, used as internal positive control, was dissolved in 20 mM Tris–HCl (pH 7.5) to a concentration of 0.82 μg⋅protein/ml in a final volume of 500 μl. All proteolytic activity assays were performed in triplicate. Addition of phenanthroline (strong inhibitor of AsES) (Chalfoun et al., 2013) to the reaction mixture was used to determine whether measured proteolytic activity in PSP1 products originated from AsES.

Proteolytic activity was calculated as the concentration of *p*NA liberated per minute using a molar extinction coefficient (𝜀_405__nm_) of 9.62 mM^−1^ cm^−1^ at 37°C and pH 7.5, where *p*NA concentration was determined spectrophotometrically at 405 nm. Autoproteolysis rate of the substrate Suc-AAPF-*p*NA was evaluated and subtracted for each measured reaction value. One unit of protease activity was defined as the release of 1 μmol of pNA per minute at 37°C and pH 7.5.

### Chemical Composition Analysis

Total protein and sugar content was analyzed for each production batch. Total proteins were determined by the Kjeldahl method, according to AOAC (2009), using a Buchi B-324 distiller. Sucrose was determined by high-performance liquid chromatography (HPLC) using a pump and autosampler Alliance. The column used for sugar separation was Sugar-Pak (Waters Technologies Corp., United States), using a 0.05 M calcium disodium EDTA solution as mobile phase at a flow rate of 0.5 ml/min and a refractive index detector (Waters Technologies Corp., United States).

### Toxicological Studies of PSP1

Toxicological testing of the PSP1 product on animals was carried out by the Argentinian company Bio Fucal S.A. according to the methodology described by the Organization for Economic Co-operation and Development (OECD) in Principles of Good Laboratory Practice and Compliance Monitoring [ENV/MC/CHEM (98) 17 OECD]. All protocols, detailed in the **Table 1**, are based on the Guide for the Care and Use of Laboratory Animals, which have been approved by Ethics Committees of Animal Care (National Academy of Sciences, Washington, 1996).

**Table 1:** Toxicity tests of the PSP1 product according to OECD guidelines.

Tier testing	Methodology	Tested dose or concentration
Acute Dermal Irritation/Corrosion Test in rabbits (*Oryctolagus cuniculus*)	OECD N° 404 (Association of Food and Drug Officials, 1959; Hobson, 1991; National Academy of Sciences, 1996; OECD, 1998)	22 μg TSP/animal
Acute Eye Irritation/Corrosion in rabbits (*O. cuniculus*)	OECD N° 405 (Association of Food and Drug Officials, 1959; Kay and Calandra, 1962; Hobson, 1991; National Academy of Sciences, 1996; OECD, 1998)	5 μg TSP/animal eye
Acute Oral Toxicity in rats (*Rattus norvegicus*)	OECD 423 (National Academy of Sciences, 1996; OECD, 1998)	225 μg TSP/kg of body weight
Acute Dermal Toxicity Test in rats (*R. norvegicus*)	OECD 402 (National Academy of Sciences, 1996; OECD, 1998)	225 μg TSP/kg of body weight
Acute Inhalation Toxicity Test in rats (*R. norvegicus*)	OECD 403 (National Academy of Sciences, 1996; OECD, 1998)	0.16 μg TSP/l of air
Dermal Sensitization Test in guinea pigs (*Cavia porcellus*)	OECD 406 (Buehler test method) (National Academy of Sciences, 1996; OECD, 1998)	40.5 μg TSP/animal
Fish Acute Toxicity (*Brachydanio rerio*)	OECD N° 203 (Finney, 1971; National Academy of Sciences, 1996; OECD, 1998)	4.5 and 0.45 μg TSP/l
Avian Acute Oral Toxicity Test (*Coturnix coturnix japonica*)	EPA. N° 712-C-96-139-OPPTS 850.2100 (National Academy of Sciences, 1996; OECD, 1998)	90 μg TSP/kg of body weight
Honeybee Oral Toxicity Test (*Apis mellifera*)	OECD N° 213 (Finney, 1971; National Academy of Sciences, 1996; OECD, 1998)	4.5 ng TSP/bee

### Plant Material and Growing Conditions

#### Strawberry

Plants of strawberry (*Fragaria × ananassa* Duch.) cv. Pájaro were obtained from the Strawberry Active Germplasm Bank (BGA) at the National University of Tucumán (San Miguel de Tucumán, Argentina). Plants were *in vitro* propagated by implanting and multiplying meristematic tissue of healthy plant runners in MS medium (Sigma) (Murashige and Skoog, 1962) and later rooted in pots containing a sterilized mixture of humus and perlite (2:1). Plants were grown in a 16-h light cycle (white fluorescent light, 350 μmol photons/m^−2^s^−1^) with 70% of relative humidity (RH) and a temperature of 28°C. Fourteen- to 16-week-old plants were used for all assays.

#### Soybean

In general, 10 seeds of soybean [*Glycine max* (L.) Merrill] from cv. A8000 RG, susceptible to soybean target spot (STS) disease, were sown into 4-L plastic pots with 3 kg of sterilized mixture of washed sieved sand, commercial humus and soil (1:1:2). Pots were immediately watered with neutral oxyquinoline sulfate (0.5 g/l). Five days after seedling emergence, each pot was thinned to three seedlings at vegetative cotyledon stage (VC), corresponding to unifoliate leaves unrolled sufficiently so that the leaf edges are not touching (Fehr et al., 1971) and remaining seedlings were regularly watered with deionized water. Plants were grown in a greenhouse under natural light conditions with controlled air temperature (mean 20.2 ± 5.2°C ranging from 12.4°C at night to 33.0°C during the day) and a RH of 82 ± 14%. In spring and autumn, high pressure sodium lamps (400 W) adjusted to a 12-h photoperiod were used to supplement natural light, giving a light intensity of approximately 220 μmol photons m^−2^s^−1^.

#### Sugarcane

Healthy single-bud sets of sugarcane (*Saccharum* spp. hybrids) variety TUCCP 77-42, susceptible to red stripe (RS), were planted in 1 L pots containing a sterile mixture of soil, debris, and sand (2:2:1). Plants were grown under greenhouse conditions (RH 60 ± 5% and temperature ranging from 24°C at night to 28 ± 2°C during the day) until use. Plants were fertilized with 120 kg/ha of Nitrodoble according to Bertani (2016), 7 days prior to PSP1-treatment.

#### Wheat

Wheat (*Triticum aestivum* L.) plant materials, ACA 304 and DM LYON, are two varieties that show differences in both baking quality and response to Fusarium head blight (FHB). Variety ACA 304 is considered as tolerant to FHB (Muller and Lassaga, 2008) whereas DM LYON, a long cycle genotype, is considered to be moderately susceptible to FHB (Donaire et al., 2016).

### Induced Resistance (IR) Against Plant Diseases

#### IR Assay Against Strawberry Anthracnose

Defense eliciting activity of PSP1 was evaluated in IR bioassays against strawberry anthracnose by a double treatment applied to plants of the strawberry cv. Pájaro as previously described by Salazar et al. (2007). First treatment consisted in spraying the youngest fully expanded leaf with the supernatant to be evaluated (coming from different production conditions or production batches), adjusted to 10 μg TSP/ml in an aqueous solution of 0.02% Tween 20 (defense-eliciting treatment). The second inoculation (pathogen challenge) was applied a week later by spraying whole foliage with a 5 × 10^6^ conidia/ml suspension of the virulent strain M11 of *Colletotrichum acutatum* J. H. Simmonds, causal agent of anthracnose in strawberry. Plants sprayed with pathogen were placed in an infection chamber at 28°C and 90% RH and 48 h later transferred to a plant growth cabinet (70% RH; 28°C and 16-h light cycle of 350 μmol photons m^−2^s^−1^) for the duration of the experiment.

##### Elicitor dose–response effect of PSP1

To study dose–response effect on disease protection, plants were initially sprayed with the PSP1 product adjusted to different total protein concentrations ranging from 0.1 to 10 μg TSP/ml (i.e., 0.1, 0.5, 1.0, 5.0, and 10.0), corresponding to a protease activity ranging from 0.05 U/ml to 5.12 U/ml. In addition, the commercial product BION 500 WG (Syngenta, Switzerland) (BION) was included as a defense-inducing control in the experiment. BION was adjusted to a final concentration of 0.5 mg/ml in an aqueous solution of 0.02% Tween 20 and applied to plant foliage 7 days prior to pathogen inoculation.

##### Experimental design in strawberry IR assays and anthracnose assessment

Disease control plants were sprayed with water (mock-treated) whereas protection control plants were sprayed with the supernatant from the SS71 culture grown statically in PDB (Chalfoun et al., 2013). Experimental design was randomized with 10 plants (biological replicates) for each treatment and the experiment was carried out twice.

Anthracnose severity was determined by the length of petiole lesions and adjudicated to the following disease severity classes: 1 (without symptoms), 2 (≤3 mm), 3 (3–10 mm), 4 (10–30 mm) or 5 (30 mm), based on the scale described by Delp and Milholland (1980).

Anthracnose disease severity index (DSI) was calculated for each plant from the scores of the petioles of each plant and each IR-treatment, and the value was expressed as a percentage using the following formula:

$$\text{Anthracnose DSI}(\%)=\sum \left(\frac{\text{A}\times 0+\text{B}\times 3+\text{C}\times 10+\text{D}\times 30+\text{E}\times 50}{\text{T}\times 50}\right)\times 100$$

where A, B, C, D, and E are the number of petioles corresponding to the numerical grade 1, 2, 3, 4, and 5, respectively, and T is the total number of petioles multiplied by the maximum severity grade 5, where T = A+B+C+D+E. An anthracnose severity of 0% was given to plants where no disease was present and 100% to plants where all petioles were assigned a score of 5.

Anthracnose DSI was registered at 7, 14, and 21 days post-inoculation (dpi) and these data were used to calculate the Area Under Disease Progress Curve (AUDPC) (Madden et al., 2007) for each plant and each IR-treatment according to the following formula:

$$AUDPC=\sum \left(\frac{X_{i}+X_{i+1}}{2}\right)\times (t_{i+1}-t_{i})$$

where *X*i corresponds to disease severity (%) at assessment *i*, *X*_i+1_ corresponds to the severity (%) at subsequent assessment *i* + 1, and (*t*_i+1_ - *t*_i_) corresponds to the number of days between the two consecutive assessments.

The AUDPC for anthracnose DSI for each treatment was compared to the AUDPC for disease control plants (AUDPC/P). Thus, a value of AUDPC/P < 1 indicated induction of resistance whereas an AUDPC/P ≥ 1 indicated that no disease control had occurred.

#### Phytopathological Test of STS Disease

##### Growth conditions of *C. cassiicola*

A pathogenic isolate of *C. cassiicola* (C4), obtained from symptomatic soybean leaves collected in the 2015 growing season at the locality of San Agustín (S 26° 49′30.43″; WO 64° 51′02.68″), Cruz Alta, Tucumán, Argentina, was used for plant inoculations. Extensive morphological and molecular identification of *C. cassiicola* isolate C4 was performed in our laboratory, which was single spore-propagated to obtain pure cultures on PDA medium supplemented with 0.2% (v/v) lactic acid under continuous fluorescent light (intensity of 165.3 μmol photons m^−2^s^−1^) at 25 ± 2°C for 12 days. Pure colonies were preserved at 4°C using Castellani’s method with distilled water during 1–20 years (Dhingra and Sinclair, 1995).

##### Inoculum preparation of *C. cassiicola*

Disks from PDA cultures of the isolate *C. cassiicola* C4 were placed on Petri dishes and incubated in a growth chamber at 25 ± 2°C with an 18-h photoperiod of fluorescent light (52.7 μmol photons m^−2^s^−1^) for 6 days. Fragments of fungal colonies were thereafter transferred to new Petri dishes and incubated under the same growing conditions for 10 days before finally being cultivated under continuous white light (165.3 μmol photons m^−2^s^−1^) for 2 days to stimulate conidia production.

Plate surface of 12-day-old fungal colonies was carefully scraped with a sterile loop and suspended in distilled sterile water. The resulting fungal suspension was shaken vigorously for 15 min at 25°C and then filtered through sterile miracloth to remove mycelial debris. Conidia were counted using a cell-counting hemocytometer (Neubauer chamber) under an optical microscope and concentration was adjusted to 5 × 10^4^ conidia/ml with a sterile solution of 0.02% Tween 20.

##### IR assay against STS

Bioassay of double treatment was performed on soybean plants at vegetative growth stage V3 corresponding to plants with three nodes on the main stem with fully developed leaves beginning with the unifoliate node (Fehr et al., 1971), following a procedure similar to that previously described in strawberry (Salazar et al., 2007). First, aerial parts of plants were sprayed to run-off with PSP1 and maintained under optimal soybean growth conditions. After PSP1-treatment, plants were inoculated by foliar spraying with a conidial suspension of the virulent isolate C4 of *C. cassiicola* (5 × 10^4^ conidia/ml). A total of 5 ml of the conidial suspension was applied per plant as a fine mist using an atomizer on the adaxial surface of leaves. After inoculation, plants were maintained in an infection chamber at 28°C, 90% RH, and with a 12-h photoperiod with fluorescent light (700 μmol photons m^−2^s^−1^). After 72 h in the infection chamber, plants were transferred to a plant growth cabinet for the duration of the experiment, where the natural photon flux density at the plant canopy height was of approximately 700 μmol photons m^−2^s^−1^, temperature held at 25 ± 2°C, and the RH was maintained at 80 ± 5% for the first 2 days using a misting system. Temperature and RH were monitored using a TH-508 thermohygrograph (Impac, Brazil).

##### Effect of PSP1 concentration and timing of application on IR against STS

To study the effect of elicitor concentration and timing of application on soybean disease resistance, IR bioassays against STS were conducted varying (i) the concentration of the PSP1 product, by adjusting the protease activity to 0.5 or 2.6 U/ml in a solution of 0.02% Tween 20, and (ii) the time elapsed between the defense-induction treatment and the pathogen challenge. Induction treatments were applied 3, 7, or 10 days before pathogen inoculation (−3, −7 and −10 days) or 3 days after (+3 days). Once application timing had been optimized with PSP1, the commercial defense-inducer BION was included in the experiment. BION in an aqueous solution of 0.02% Tween 20 (0.5 mg/ml) and a purified AsES solution (0.2 μg/ml), were both applied to plant foliage 3 days prior to pathogen inoculation.

##### Experimental design in soybean IR assays and STS assessment

Normal disease development was monitored in pathogen control plants, firstly sprayed with water (mock) and then inoculated with the pathogenic strain C4 of *C*. *cassiicola* (P). Experimental design was randomized with nine plants (biological replicates) for each treatment and each experiment was carried out twice. STS severity was evaluated on the third and fourth trifoliate leaves, corresponding to soybean growth stages V3 and V4, at 4, 7, and 10 dpi using a standard area diagram set (Soares et al., 2009). Covered lesion areas (%) were adjudicated to the following disease severity classes: 1 (0–1%), 2 (2–10%), 3 (11–20%), 4 (21–50%) or 5 (≥50%).

DSI of STS (STS DSI) was calculated for each plant from the scores of the six leaflets of each plant and for each IR-treatment, and the value was expressed as a percentage using the of following formula:

$$\text{STS DSI}(\%)=\sum \left(\frac{\text{A}\times 0+\text{B}\times 2+\text{C}\times 11+\text{D}\times 21+\text{E}\times 50}{\text{T}\times 50}\right)\times 100$$

where A, B, C, D, and E are the number of leaflets corresponding to the numerical grade 1, 2, 3, 4, and 5, respectively, and T is the total number of leaflets multiplied by the maximum severity grade 5, where T = A+B+C+D+E. A STS severity of 0% was given to plants where no disease was present, and 100 to plants where all leaflets were assigned a score of 5%.

Data from the STS DSI evaluation were used to calculate the AUDPC (Madden et al., 2007) for each plant and each IR-treatment according to the formula described above (Shaner and Finney, 1977). The AUDPC for STS DSI for each IR-treatment was relativized to the AUDPC for pathogen control plants (AUDPC/P). Thus, a value of AUDPC/P < 1 indicated induction of resistance whereas an AUDPC/P ≥ 1 indicated that no disease control had occurred.

#### IR Assay Against Sugarcane RS Disease Under Controlled Conditions

Sugarcane assays were performed following the procedure reported by Jones et al. (2001) with some modifications. PSP1 and water (mock) were applied 5 and 2 days before pathogen inoculation. PSP1 adjusted to 5.1 U/ml (protease activity) was applied by spraying plants to run-off. In addition, the product BION was included as a defense-inducing control in the experiment and applied to plant foliage as an aqueous solution adjusted to 0.5 mg/ml with 0.02% Tween 20 5 days prior to pathogen inoculation. For pathogen infection, a virulent isolate of *A. avenae* was grown on nutrient broth (NB) until exponential phase and a 10^8^ cfu/ml suspension was prepared based on spectrometric absorbance, according to Bertani (2016). The inoculum was applied by spraying both the adaxial and abaxial surfaces of sugarcane leaves until run-off with a manual atomizer. Infected plants were kept at 30°C and 100% RH (covered with a plastic bag) for the first 24 h, and then a >80% RH was maintained until the end of the IR assay.

##### Experimental design in sugarcane IR assays and RS assessment

Mock-treated plants were used as disease control. Plants were arranged in a randomized complete block design and 16 sugarcane plants (biological replicates) were used for each treatment. RS severity was evaluated on all leaves of each plant at 5 and 9 dpi according to the International Society of Sugar Cane Technologists scale ranging from 0 (no disease) to 9 (more than 50% of the foliar area affected by the disease) (Bertani et al., 2015; Bertani, 2016). The covered lesion areas (%) were adjudicated to the following disease severity classes: 0 (0%), 1 (<0.5%), 2 (∼0.5%), 3 (∼1%), 4 (∼5%), 5 (∼10%), 6 (∼25%), 7 (∼35%), 8 (∼50%), or 9 (>50%).

Disease index of RS (RS DI) was calculated for each plant from the scores of the leaves of each plant for each IR-treatment using the formula below.

$$\text{RS DI}=\sum \left(\frac{\begin{array}{c} \text{A}\times 0+\text{B}\times 1+\text{C}\times 2+\text{D}\times 3+\text{E}\times 4+\\ \text{F}\times 5+\text{G}\times 6+\text{H}\times 7+\text{I}\times 8+\text{J}\times 9 \end{array}}{\text{T}}\right)$$

where A, B, C, D, E, F, G, H, I, and J are the number of leaves corresponding to the numerical grade from 0 to 9, respectively, and T is the total number of leaves, where T = A+B+C+D+E+F+G+H+I+J.

### Statistical Analysis of IR Assay Data

Statistical analyses were performed using the software Infostat (Di Rienzo et al., 2015). In IR assays against anthracnose or STS diseases, treatment effects were assessed by Analysis of Variance (ANOVA). Factorial ANOVA test was done with data of two assays, considering experiment, treatment and their interaction as main effects. Means of DSI at each time and/or AUDPC (AUDPC/P) values were compared among treatments by Tukey’s HSD test at the 0.05 significance level and grouping was indicated with letters (**Tables 2–4** and **Figures 2**, **3**). A bilateral Dunnett’s test (*P*-value ≤ 0.05) was used to compare each treatment to disease control treatment and significant differences between both were indicated with bold letters (**Tables 2–4** and **Figures 2**, **3**). In IR assays against RS disease, treatment effects were assessed by General Lineal Mixed Models. Means of DI at each time were compared among treatments by LSD Fisher test at the 0.05 significance level and grouping was indicated with letters (**Figure 4**).

**Table 2:** Protein yield and plant defense-eliciting activity of supernatants derived from *A. strictum* SS71 cultures under different fermentation conditions, supernatant processing and storage.

Culture medium and growing conditions	Fermentation scale and time	Processing and conditioning	Storage temperature	Protein yield (μg TSP/ml)	AUDPC/P
Static culture in PDB under fluorescent light	0.25 L for 21 days	Ce Fi	4°C	9.87	0.46 ± 0.05 **a**
Culture in SMB with	0.25 L for 7 days	Ce Fi	4°C	31.01	0.50 ± 0.04 **a**
agitation in darkness	1.5 L for 3 days	Ce pH 5.5 Fi	4°C	44.70	0.61 ± 0.11 **a**
			37°C for 6 months	44.33	0.56 ± 0.06 **a**
			45°C for 6 months	26.02	0.57 ± 0.09 **a**

*Total extracellular soluble protein (TSP) concentration was measured immediately before foliar application of supernatants. Supernatant-treated and mock-treated plants were inoculated with a pathogenic strain of C. acutatum 7 days after application. Ten biological replicates were assessed for each treatment and the experiment was carried out thrice. Factorial ANOVA test indicated no significant differences among the three experimental repetitions (P > 0.05) and results from one representative experiment are shown. Anthracnose disease development in strawberry plants was determined as AUDPC from the disease severity values at different time points. Mean and standard error (SE) values of AUDPC/P (AUDPC relativized to the AUDPC mean value for pathogen control treatment) are reported for each treatment calculated from one representative experiment with 10 biological replicates. Ce, centrifugation (9000 × g, 15 min); pH 5.5, pH adjustment with HCl; Fi, membrane filtration (0.22 μm). A value of AUDPC/P < 1 indicates induced resistance to anthracnose and an AUDPC/P ≥ 1 indicates no protection. Values followed by different letters are significantly different according to Tukey’s HSD test (P < 0.05), where AUDPC/P value of pathogen treatment is 1 and its class is b. Bold letters indicate statistically significant differences in disease control as compared to anthracnose development of M11-infected mock-treated strawberry plants (Dunnett’s test; P < 0.05).*

**Table 3:** Correlation between proteolytic activity and elicitor-induced plant defense activity in different batches of PSP1 production.

Treatment	Year	TSP (μg/ml)	Protease activity (U/ml)	Specific protease activity (U/mg protein)	AUDPC/P
AsES	–	–	–	–	0.40 ± 0.11 **a**
PSP1 batch A	2014	59.20	0.54	9.16	0.52 ± 0.05 **a**
PSP1 batch B	2014	60.11	4.71	7.84	0.50 ± 0.04 **a**
PSP1 batch C	2015	17.10	**0.015**	0.88	1.02 ± 0.05 b
PSP1 batch D	2015	42.75	15.57	36.35	0.45 ± 0.04 **a**
PSP1 batch E	2015	41.36	**0.016**	0.38	1.03 ± 0.07 b
PSP1 batch F	2015	48.23	24.67	51.15	0.52 ± 0.05 **a**
PSP1 batch G	2015	32.90	25.78	78.36	0.43 ± 0.06 **a**
PSP1 batch H	2016	38.66	29.45	76.18	0.88 ± 0.05 **ab**
PSP1 batch I	2016	46.93	8.91	18.97	0.82 ± 0.09 **ab**
BION	–	–	–	–	0.36 ± 0.09 **a**

*The purified protein AsES (0.2 μg/ml) and commercial product BION (0.5 mg/ml) were included as positive controls of defense-induction in the experiment. Letters A to I correspond to 9 individual fermentation batches of PSP1. Batches showing no protease activity are highlighted in bold. Ten biological replicates (potted plants) were assessed for each treatment and the experiment was carried out thrice. Factorial ANOVA test indicated no significant differences among the three experimental repetitions (P > 0.05) and results from one representative experiment are shown. Anthracnose disease development in strawberry plants was determined as AUDPC from the disease severity values at different time points. Mean and standard error (SE) values of AUDPC/P (AUDPC relativized to the AUDPC mean value for pathogen control treatment) are reported for each treatment calculated from one representative experiment with 10 biological replicates. Values followed by different letters are significantly different according to Tukey’s HSD test (P < 0.05), where AUDPC/P value of pathogen treatment is 1 and its class is b. Bold letters indicate statistically significant differences in anthracnose protection as compared to mock-treated strawberry plants infected with the pathogenic strain M11 (Dunnett’s test; P < 0.05).*

**Table 4:** Dose-dependent evaluation of PSP1-eliciting activity against anthracnose development in strawberry.

Treatment	TSP (μg/ml)	Protease activity (U/ml)	AUDPC/P
PSP1	10.0	5.12	0.52 ± 0.05 **a**
	5.0	2.56	0.54 ± 0.03 **a**
	1.0	0.51	0.51 ± 0.04 **a**
	0.5	0.26	0.48 ± 0.05 **a**
	0.1	0.05	0.95 ± 0.04 b
BION	–	–	0.36 ± 0.09 **a**

*A single PSP1 batch (F) diluted to concentrations ranging from 5.12 to 0.05 U/ml of protease activity was applied on strawberry plants that were thereafter challenged with C. acutatum strain M11. Commercial product BION (0.5 mg/ml) was included as a positive control of defense-induction in the experiment. Ten biological replicates (potted plants) were assessed for each treatment and the experiment was carried out thrice. Factorial ANOVA test indicated no significant differences among the three experimental repetitions (p > 0.05) and results from one representative experiment are shown. Anthracnose disease development in strawberry plants was determined as AUDPC from the disease severity values at different time points. Mean and standard error (SE) values of AUDPC/P (AUDPC relativized to the AUDPC mean value for pathogen control treatment) are reported for each treatment calculated from one representative experiment with 10 biological replicates. Values followed by different letters are significantly different according to Tukey’s HSD test (P < 0.05), where AUDPC/P value of the pathogen treatment is 1 and its class is b. Bold letters indicate statistically significant differences in anthracnose protection of inducer-treated plants as compared to mock-treated strawberry plants, both infected with the anthracnose virulent strain M11 (Dunnett’s test; P < 0.05).*

### Detection of Defense Responses in Soybean

Defense-eliciting activity of PSP1 and BION was evaluated in healthy soybean plants cv. A8000 RG at growth stage V3 by analyzing occurrence of oxidative burst and defense-related gene expression. Treatments were: PSP1 adjusted to a protease activity of 0.5 U/ml and supplemented with 0.02% Tween 20; BION prepared at a concentration of 0.5 mg/ml (as recommended by the manufacturer) with addition of 0.02% Tween 20; and mock (sterile solution of 0.02% Tween 20).

#### Oxidative Burst Analysis

**FIGURE 1:**
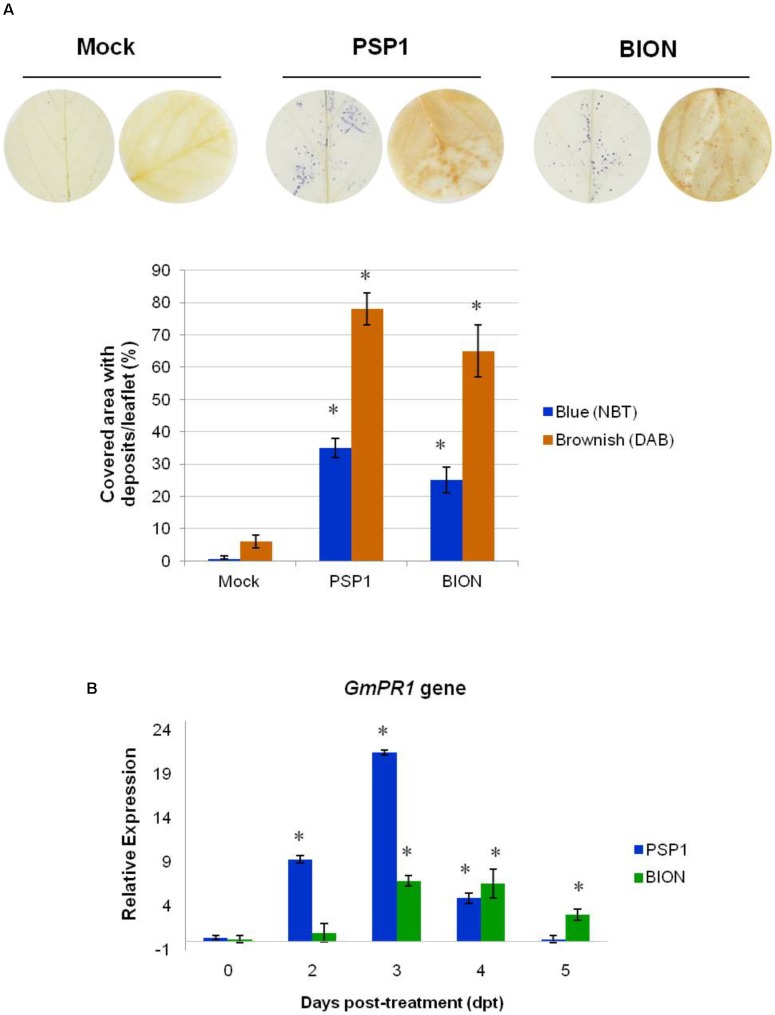
Induction of defense responses by PSP1 and BION treatments in soybean plants. **(A)** Accumulation of superoxide (${\text{O}}_{2}^{•-}$) and hydrogen peroxide (H_2_O_2_) was analyzed in foliar tissue of plants treated with mock, PSP1 (0.5 U/ml) and BION (0.5 mg/ml) 4 h post-treatment by NBT and DAB staining methods, respectively. Twenty biological replicates (leaflets) were assessed for each treatment and the experiment was carried out twice. Factorial ANOVA test indicated no significant differences between the two experimental repetitions (*p* > 0.05) and results from one representative experiment are shown. Quantification of ROS accumulation was performed by measurement of the foliar area with blue (${\text{O}}_{2}^{•-}$) and brownish (H_2_O_2_) deposits with ImageJ software, and expressed as covered area/leaflet. Mean and standard error (SE) values of covered area/leaflet are reported for each treatment calculated from one representative experiment with 20 biological replicates. Asterisks indicate significant differences with respect to mock-sprayed control plants according to Dunnett’s test (*p* < 0.05). Each photo shows two representative leaf disks from 20 leaflets analyzed for each treatment 4 h after application. **(B)** Time course of quantitative expression of gene *GmPR1* in response to treatment of soybean plants with PSP1 and BION. Leaf samples of plants treated with mock, BION and PSP1 were collected at 0, 2, 3, 4, and 5 dpt. Two biological replicates (obtained from a poll of leaflets from three plants) were tested thrice (technical replicates) for each treatment and the experiment was carried out twice. Relative expression levels were calculated using the expression of the soybean beta-tubulin (*GmTUBB2*) gene as non-variable constitutive expression for normalization of values. Expression data were calibrated against mock-treated plants sampled at each time point. Data from the two experiments were considered in the analysis. For each time point, mean levels of relative expression and SE are reported for each treatment calculated from a pool of two experiments with two biological replicates assessed thrice (technical replicates). Asterisks indicate significant differences with respect to mock-sprayed control plants, calculated by fgStatistics software (*P* < 0.05).

Accumulation of reactive oxygen species (ROS) was detected by specific *in situ* histochemical staining procedures. The generation of superoxide radical ion (${\text{O}}_{2}^{•-}$) was detected according to Doke (1983) trough superoxide-dependent reduction of the compound nitroblue tetrazolium (NBT) with formation of a blue precipitate. The production of hydrogen peroxide (H_2_O_2_) was detected by reaction with 3,3′-diaminobenzidine (DAB) generating a brownish precipitate according to Thordal-Christensen et al. (1997). Mock- BION- and PSP1-treated leaflets were harvested 4 h after foliar application and stained with DAB or NBT as described previously (Chalfoun et al., 2011). Twenty trifoliate leaves (biological replicates) collected from different plants were analyzed per treatment and each experiment was carried out twice. Quantification of brownish (H_2_O_2_) and blue (${\text{O}}_{2}^{•-}$) deposits was carried out using ImageJ software and an area value was obtained. Results were expressed as area (%) covered with brownish/blue deposits in each PSP1-treated leaflet. Standard error (*SE*) was calculated for each time point as: SE = $\frac{SD}{\sqrt{n}}$, where *SD* is the standard deviation of data values from its mean and *n* is the number of biological replicates. Statistical analysis was carried out using software Infostat (Di Rienzo et al., 2015). Treatment effects about covered area (%) were assessed by Analysis of Variance (ANOVA). Factorial ANOVA test was done with data of the experimental repetitions, considering experiment, treatment and their interaction as main effects. Mean of covered area from each treatment was compared to mock- treatment by bilateral Dunnett’s test (*P*-value ≤ 0.05) and significant differences were indicated with asterisks (**Figure 1A**).

#### RNA Extraction and q-PCR Analysis

Specific gene expression analysis was performed on the youngest totally expanded trifoliate leaf at 0, 1, 2, 3, 4, and 5 days post-treatment (dpt). Leaves harvested from three individual plants were pooled, weighted and immediately frozen in liquid nitrogen and kept at −80°C until use. Total plant RNA was isolated from 100 to 200 mg of leaf tissue using Trizol Reagent (Invitrogen, United States) and the resulting extracted RNA was then treated with DNase I (Thermo Fisher, United States) to remove possible contaminant genomic DNA. The concentration and purity of all samples was measured in a picodrop Model VersaWave (Expedeon, United Kingdom). RNA (1 μg) was retro-transcribed into cDNA according to manufacturer’s indications (Thermo Fisher Scientific, United States) and final cDNA concentration was adjusted to 25 ng/μl with sterile Milli-Q water (NW Ultrapure Water System, China). Quantitative polymerase chain reaction (q-PCR) was performed using an iQ Supermix SYBRGreen (Bio-Rad Laboratories Inc., United States) and a Mini Optic on Real Time PCR System equipment (Bio-Rad Laboratories Inc., United States). Reaction mixture for q-PCR was prepared as follows: 1 μl of each primer (5 μM), 5.0 μl cDNA (25 ng/μl), 12.5 μl SYBRGreen and 5.5 μl sterile Milli-Q water. The real-time PCR program used consisted in a heating step at 95°C for 1 min, followed by 40 cycles of 85 s (95°C for 15 s, 64°C for 30 s and 72°C for 40 s). Gene expression values were normalized using soybean beta-tubulin (S-beta-2) gene (*GmTUBB2*; GenBank accession number M21297.1) (Guiltinan et al., 1987) as a stable reference gene for *G. max*, which was amplified with the primers AATGCGTGAGAGCCTTCACA (Fw) and TGGCGCCGATCTGGTT (Rv). The primers used to analyze soybean pathogenesis-related protein 1-like gene (*GmPR1*; GenBank accession number XM_003545723.3) expression were CACAACGCTGCAAGATCACA (Fw) and GCGACTGCGTTATCCCAAAC (Rv). The q-PCR data and primer efficiencies were analyzed using the LinReg PCR software (Ruijter et al., 2009). Gene expression data was automatically normalized according to the reference gene by the algorithm developed by Pfaffl (2001). Two biological replicates, each one obtained by pooling one trifoliate leaf from each of three individual plants, were used for each time point. Three technical replicates were used for each gene analyzed in each time point. *GmPR1* gene expression was determined as the ratio between the expression levels of mock-treated plants and those treated with PSP1 or BION and SE was calculated for each time point according to the formula described above. Differences between means in qPCR data were analyzed using fgStatistics software interface (Di Rienzo, 2009) (*P*-value < 0.05) and are indicated with asterisks (**Figure 1B**).

### Field IR Trial Against Wheat FHB

Field trial was performed during two consecutive growing seasons, 2013 and 2014, in the locality of Azul (S 36° 48′; WO 59° 51′), in the Province of Buenos Aires, using the two wheat varieties, ACA 304 and DM LYON. Field trial for each genotype was conducted with a split-plot design with seven blocks where each one was divided in two plots, one corresponding to control- and the other to PSP1- treatment with an individual plot size of 0.85 m^2^. Trials received herbicide applications according to standard wheat crop management.

A double aerial application of PSP1 adjusted to 0.5 U/ml (protease activity) was carried out by spraying plants to run-off at growth stage Z2.1-2.3 (early tillering) and subsequently at stage Z3.1-3.3 (stem elongation). For inoculum preparation, a virulent isolate of *F. graminearum* was grown for 10 days on liquid medium containing (g/l): 1.0 NH_4_NO_3_, 1.0 KH_2_PO_4_, 0.5 MgSO_4_.7H_2_O, 1.0 yeast extract and 15.0 carboximethyl cellulose, supplemented with 0.2 g/l streptomycin. All PSP1- and mock-treated plants were inoculated with the pathogen at stage Z6.5 (anthesis) by spraying an aqueous suspension of 1.0 × 10^4^ conidia/ml until run-off (Akinsanmi et al., 2004, 2007).

Mock-treated plants were used as positive disease control. Disease development was estimated visually for 20 spikes randomly collected from each plot at 20 dpi (Campbell and Lipps, 1998). Incidence was recorded as the proportion of diseased spikes (number of infected spikes divided by the total number of spikes sampled). Severity was recorded as the average proportion of diseased spikelets per spike (sum of the proportion of diseased spikelets per spike divided by the total number of spikes sampled). Once physiological maturity was reached, spikes were harvested manually from each plot and 1000-seed weight (TSW) and germination rate (GR) were determined. GR was determined by placing 50 seeds collected from infected plants on moistened filter paper and was expressed as percentage of germinated seeds after 7 days of incubation.

#### Data Analysis of Wheat Field Trial

Treatment effects were assessed by Analysis of Variance (ANOVA). Means were compared by Tukey’s HSD test (*P*- value ≤ 0.05) and significant differences for the different traits were indicated with letters (**Table 6**).

## Results

### Optimization of the Fermentation Process of *A. strictum* SS71 and Conditioning of the Supernatant to Maximize the Plant Defense-Eliciting Activity

To develop a robust and efficient laboratory scale fermentation process of *A*. *strictum* SS71, fungal growth and TSP were compared among cultures obtained from two different growth media and incubated under different growth conditions. Fungal growth was initially slow for all growth conditions and media tested, but when SS71 was grown with agitation in SMB, all glucose was consumed within 7 days, while the fungus needed up to 21 days for glucose depletion when grown statically in PDB. Although both fermentation methods generated fungal growth, differences in cultural appearance were detected as SS71 grown with agitation generated pellets with shorter and more branched hyphae as compared to cultures of static growth with a compact biomass on the medium surface.

Total soluble protein content of the supernatant (containing the AsES elicitor) varied noticeably between the two fermentation conditions. SS71 grown with agitation in SMB containing 1% glucose rendered 31.01 μg TSP/ml after 7 days of incubation, whereas the supernatant of a SS71 culture in static PDB with 2% glucose only yielded 9.87 μg TSP/ml after 21 days of growth. In conclusion, TSP yield increased approximately threefold when SS71 grown with agitation in a much shorter fermentation time and from less glucose dissolved in the medium. Once the most favorable fermentation condition, as determined by extracellular TSP production, had been established, a small scale-up of the procedure was developed. Interestingly, when the fermentation batch volume was increased, glucose consumption was quicker and completed already after 72 h accompanied by an even higher TSP yield (>40 μg TSP/ml) (**Table 2**).

To determine defense-eliciting activity of individual supernatants from fungal fermentation batches, protection against anthracnose disease in strawberry plants first treated with the SS71 supernatant to be tested and subsequently infected with the virulent *C. acutatum* strain M11 was evaluated. **Table 2** shows that strawberry plants treated with supernatants from SS71 cultures exhibited significant reduction (40–60%) of anthracnose development as compared to mock-treated pathogen-infected control plants (AUDPC/P). To be able to store the same fermentation batch for longer periods of time, fungal growth and microbe contamination had to be controlled while maintaining the defense-inducing capability of the supernatant. Therefore, acidification of the fermentation broth was incorporated as an additional step in the downstream processing between centrifugation (separation of cell mass and debris from the liquid) and filtration (elimination of residual microorganisms). Additionally, this corresponding acidified axenic liquid product was found to be storable at 45°C for at least 6 months maintaining its original plant defense-eliciting ability (**Table 2**). The resulting supernatant was named PSP1, as a potential plant protection and stimulation product based on compounds of biological origin, and used for all further experiments in this study. Further analysis of the chemical composition of PSP1 indicated that 1 L of a standard production batch contained 114 mg of total protein (total Nitrogen) and low levels of sucrose (<1 g).

### Relationship Between Protease Activity and Induction of Plant Defense Response

When testing various individual samples originating from different SS71 fermentation batches, we found that plant defense-eliciting activity determined by the anthracnose protection bioassay in strawberry plants and expressed as AUDPC/P, exhibited some variability and that these differences did not always correlate with the TSP concentration (**Table 3**). Therefore, several batches of PSP1 were tested for *in vitro* protease activity and compared to their pathogen defense-eliciting activity. These studies showed a positive correlation between subtilisin-like protease activity and induction of plant defense response in all PSP1 batches tested. Batches with protease activity demonstrated a reduction in disease symptoms ranging from 30 to 60% in comparison with mock-treated disease control plants whereas the two batches lacking protease activity did not trigger protection against disease development (**Table 3**). This result demonstrated that the proteolytic activity of the supernatant can be used as an indicator of defense-induction activity and as a direct result of these studies all fermentation batches of SS71 (PSP1) were checked for proteolytic activity before used in any plant protection experiment. Commercial product BION and the protein AsES were included as positive controls for defense-induction and gave a disease protection comparable to PSP1 batches.

### Dose–Response Effect in Defense-Eliciting Activity of PSP1

After a correlation between protease activity and plant defense-induction of PSP1 had been established, the minimum protease activity for elicitor activity was determined in protection assays against strawberry anthracnose. For this experiment the same PSP1 production batch, F, diluted to different concentrations was tested. Results presented in **Table 4** showed that as the protease activity of PSP1 was step-wise reduced, its elicitor activity was kept until reaching an enzymatic activity of 0.26 U/ml. It is interesting to notice that no clear dose–response effect of the defense-eliciting activity against anthracnose in strawberry was observed, as there was no significant difference in disease protection between the highest protease activity tested (5.12 U/ml) and the 20-fold lower level (0.26 U/ml; **Table 4**). This concentration range of PSP1 induced a protection against anthracnose comparable to the product BION.

### Environmental Toxicology and Non-target Effects of PSP1 Formulation

The PSP1 formulation is intended to be used as a sustainable crop disease management product by itself or/and as a component of Integrated Pest Management (IPM) programs. To be able to incorporate PSP1 as a registered product in a reduced pesticide system, the compatibility of this product with non-target organisms such as insects, fish and birds was studied. Furthermore, toxicity studies of the elicitor PSP1 product on different mammalian species (rats, rabbits, and guinea pigs) were performed. All tests (**Table 5**) showed that the formulated product of PSP1 was harmless to beneficial organisms and non-toxic to mammalian species at concentrations 50 times higher than those used in plant experiments.

**Table 5:** Toxicological studies of PSP1 applied in high doses on bee, fish, chicken and three mammalian species (rat, rabbit, and guinea pig).

Tier testing	Tested dose or concentration	Effects	IOBC classification
Acute Dermal Irritation/Corrosion Test in rabbits	22 μg TSP/animal	0	
Acute Eye Irritation/Corrosion in rabbits	5 μg TSP/animal eye	0	
Acute Oral Toxicity in rats	225 μg TSP/kg of body weight	LD_50_ > 225 μg TSP/kg of body weight	
Acute Dermal Toxicity Test in rats	225 μg TSP/kg of body weight	LD_50_ > 225 μg TSP/kg of body weight	
Acute Inhalation Toxicity Test in rats	0.16 μg TSP/l of air	LD_50_ > 0.16 μg TSP/l of air	
Dermal Sensitization Test in guinea pigs	40.5 μg TSP/animal	0	
Fish Acute Toxicity	4.5 and 0.45 μg TSP/l	LD_50_ > 4.5 μg TSP/l at 96 h	Practically harmless
Avian Acute Oral Toxicity Test	90 μg TSP/kg of body weight	LD_50_ > 90 μg TSP/kg of body weight to 14 days	Practically harmless
Honeybees Oral Toxicity Test	4.5 ng TSP/bee	LD_50_ > 4.5 ng TSP/bee at 48 h	Virtually harmless

*All toxicity tests were carried out according to OECD guidelines and IOBC standard protocols. LD_50_, median lethal dose.*

### Defense Responses Induced by PSP1 in Soybean

To verify that pathogen defense responses could be induced by PSP1 in soybean, in a similar way to strawberry (Chalfoun et al., 2013) and Arabidopsis (Hael-Conrad et al., 2015), oxidative burst by accumulation of superoxide radical (${\text{O}}_{2}^{•-}$) and hydrogen peroxide (H_2_O_2_) and defense gene expression were analyzed. The pathogenesis-related gene *GmPR1*, induced by SA, was chosen as genetic marker of induced defense response. As positive control and a good comparison the commercial defense inducer known in soybean BION, was included in experiments.

As shown in **Figure 1A**, foliar application of PSP1 induced a strong accumulation of ${\text{O}}_{2}^{•-}$ and H_2_O_2_ in plant leaf tissue 4 h after treatment. A similar result was observed for BION-treated leaves, whereas no ${\text{O}}_{2}^{•-}$ or H_2_O_2_ were detected in mock-treated plants (**Figure 1A**). In addition, *GmPR1* expression was induced by PSP1 with a significant up-regulation already 2 dpt, reaching a maximum 21-fold induction 3 dpt, before returning to basal expression levels 5 dpt. A similar expression pattern of *GmPR1*, although not as strong, was observed in BION-treated leaves, but with a slight delay compared to PSP1-treated plants, as induction started and reached the maximum level 3 dpt and was maintained until 5 dpt (**Figure 1B**). These results clearly demonstrated that foliar application of PSP1 induced pathogen defense responses in soybean.

### Evaluation of Protection Against STS Disease in Soybean by Foliar Application of PSP1

**FIGURE 2:**
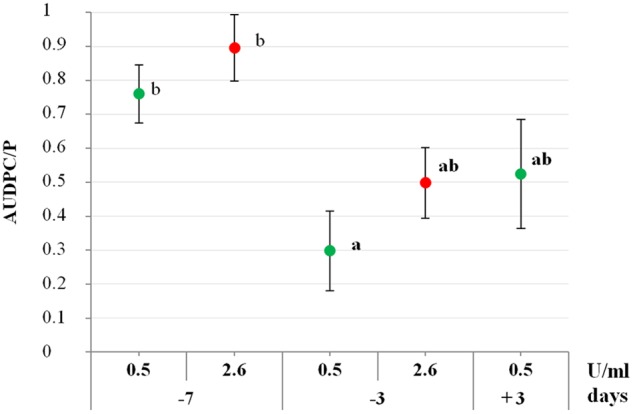
Evaluation of the defense-eliciting activity of PSP1 against soybean target spot (STS). Two different protease concentrations (0.5 and 2.6 U/ml) were applied to plants grown under controlled conditions at different time intervals before and after pathogen inoculation and induced resistance against STS was determined. Nine biological replicates (potted plants) were evaluated for each treatment and the experiment was carried out twice. Factorial ANOVA test indicated no significant differences between the two experimental repetitions (*P* > 0.05) and results from one representative experiment are shown. STS disease development in soybean plants was determined as AUDPC calculated from the disease severity values at different time points. Mean and standard error (SE) values of AUDPC/P (AUDPC relativized to the AUDPC mean value for pathogen control treatment) are reported for each treatment calculated from one representative experiment with nine biological replicates. Values followed by different letters are significantly different according to Tukey’s HSD test (*P* < 0.05), where AUDPC/P value of pathogen treatment is 1 and its class is b. Bold letters indicate statistically significant differences in STS protection of PSP1-treated plants as compared to mock-treated soybean plants, both infected with pathogenic strain C4 (Dunnett’s test; *P* < 0.05).

To assess if PSP1 not only induced defense response in soybean, but also protected plants against disease development, an IR-assay against *C*. *cassiicola*, causal agent of STS, was developed. To optimize the bioassay, different time intervals between defense-induction treatment and pathogen inoculation were assessed using two different concentrations of PSP1 from the same production batch (F), corresponding to 0.5 and 2.6 U/ml of protease activity. Soybean plants sprayed with the lower concentration of PSP1 (0.5 U/ml) 3 days before pathogen inoculation (−3) exhibited a significant reduction in STS development compared with mock-treated plants (AUDPC/P; **Figure 2**). In contrast, plants treated with the same PSP1 concentration (0.5 U/ml) 7 days prior to pathogen inoculation (−7) demonstrated a much lower reduction in STS development (**Figure 2**).

Additionally, to determine if an increase of the PSP1 concentration could improve plant protection, PSP1 with a fivefold higher concentration (2.6 U/ml) was applied 3 or 7 days before pathogen inoculation. Results showed that treatment with the higher concentration of PSP1 mimicked the lower concentration, showing little or no reduction in STS development when applied at −7 days whereas a good protection response was observed when applied at −3 days (**Figure 2**). Interestingly, plants sprayed with the PSP1 solution adjusted to 0.5 U/ml 3 days after pathogen infection (+3) showed a protective effect against disease development, similar to plants treated at −3 days. Furthermore, none of the two PSP1 concentrations used in the experiment showed any phytotoxic effect on soybean or antifungal activity against the virulent strain of *C. cassiicola* *in vitro* and *in situ* on the foliar surface (data not shown). In conclusion, a PSP1 concentration corresponding to 0.5 U/ml of protease activity is sufficient to produce an IR response against STS in soybean plants when applied 3 days before or after pathogen inoculation.

### Comparison of Defense-Eliciting Activity Between PSP1 and BION in Soybean

**FIGURE 3:**
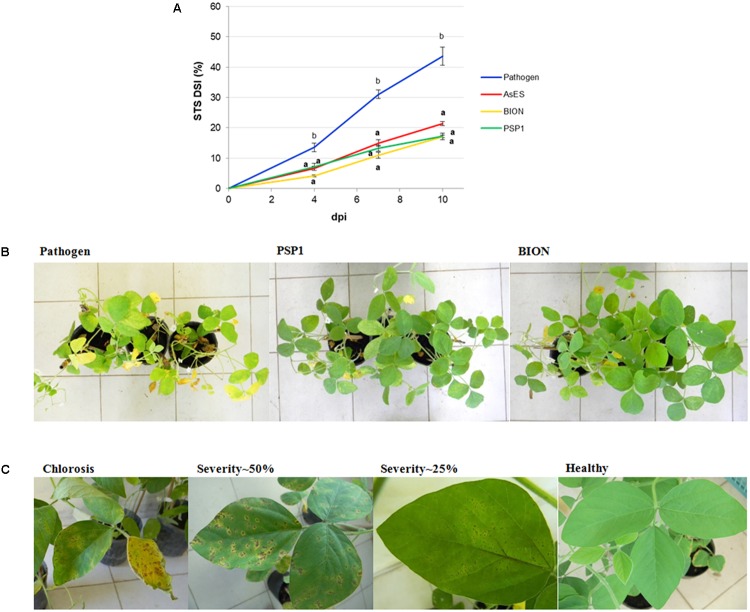
Target spot disease development in soybean plants pre-treated with PSP1, purified AsES and BION. Aqueous solutions of PSP1 (0.5 U/ml), purified protein AsES (0.2 μg/ml) or BION (0.5 mg/ml) were sprayed on soybean plants grown under controlled conditions 3 days prior to inoculation with the virulent strain C4 of *C. cassiicola* and induced resistance against STS was determined. Disease control treatment corresponded to mock-treated plants infected with the pathogen. **(A)** Nine biological replicates (potted plants) were assessed for each treatment and the experiment was carried out twice. Factorial ANOVA test indicated no significant differences between the two experimental repetitions (*P* > 0.05) and results from one representative experiment are shown. STS severity was expressed as the percentage of foliar surface affected with disease symptoms and calculated as disease severity index (DSI) at 4, 7, and 10 dpi. STS disease development in soybean plants was determined as AUDPC from the disease severity values at different time points. Mean and standard error (SE) values of DSI at different time points and AUDPC are reported for each treatment calculated from one representative experiment with nine biological replicates. Values followed by different letters are significantly different according to Tukey’s HSD test (*P* < 0.05). Bold letters indicate statistically significant differences in STS protection of inducer-treated plants as compared to mock-treated soybean plants, both infected with pathogenic strain C4 (Dunnett’s test; *P* < 0.05). **(B)** Pictures show the top view of whole plant sets treated with mock, BION and PSP1 at 10 days after *C*. *cassiicola* pathogen inoculation. One representative picture is used to illustrate results for each treatment. **(C)** Typical STS symptoms are shown for individual leaves representative from pathogen control plants (severity∼50% and chlorosis) and those pre-treated with PSP1 or BION (healthy trifoliate leaf and severity∼25%).

To test the efficiency of the PSP1 technology in soybean, its protection effect against STS disease was compared with the commercial product BION. As the best protection of soybean against *C. cassiicola* infection was observed in plants sprayed with a PSP1 solution (0.5 U/ml of protease activity) 3 days prior to pathogen inoculation (as shown previously in **Figure 2**), these parameters were used for evaluating disease resistance induced by all treatments. BION was prepared to the concentration recommended by the manufacturer for disease control in leguminous crops. Results demonstrated that treatments of soybean with PSP1, BION or AsES exhibited significant protection against STS development as compared to inoculated mock-treated control plants, and that no statistically significant difference was observed among severity values of the three treatments (**Figure 3A**), indicating that PSP1 treatment conferred a similar STS disease control as the purified protein AsES and the commercial product BION. **Figure 3B** shows mock-, BION-, or PSP1-treated plants 10 days after infection with *C. cassiicola*. Both PSP1 and BION showed good protection as demonstrated by healthy and vigorous plants with only a few leaves developing necrotic spots symptomatic for the disease, covering as maximum a 25% of foliar surface as exemplified by leaves in **Figure 3C**. In contrast, mock-treated plants showed severe overall necrosis, chlorosis and defoliation and most leaves exhibited over 50% coverage of STS symptoms (**Figure 3C**).

### Disease Protection of PSP1 Application in Sugarcane and Wheat

To further evaluate the potential disease protection capability of PSP1 in other crop species, IR assays against RS disease in sugarcane caused by the bacterial pathogen *A. avenae*, and FHB in wheat caused by the necrotrophic fungi *F. graminearum* were analyzed. For the wheat experiment two genotypes with different behavior against the disease (susceptible and moderate resistant), were included in experiments.

**FIGURE 4:**
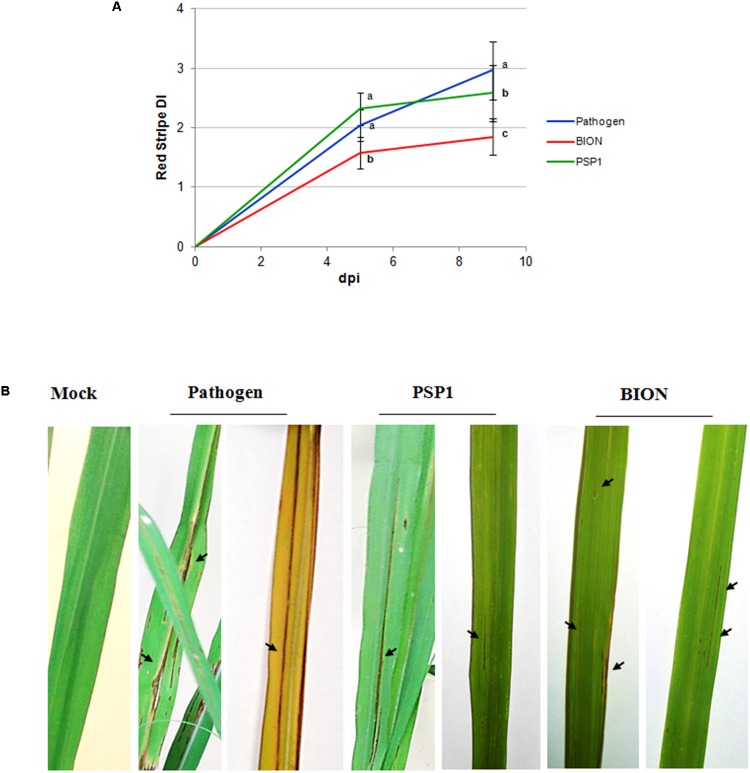
Development of red stripe (RS) disease in sugarcane after treatment with PSP1 and BION. **(A)** RS disease development was evaluated in sugarcane plants (variety TUCCP 77-42) treated by foliar application of PSP1 (5.1 U/ml) or BION (0.5 mg/ml) and thereafter inoculated with a virulent strain of the bacterial pathogen *A. avenae*. A total of 16 biological replicates (potted plants) were assessed for each treatment and the experiment was carried out once. RS severity was assessed as the foliar surface affected with disease symptoms at 5 and 9 dpi and disease index (DI) was calculated. Mean and standard error (SE) values of DI at different time points are reported for each treatment calculated from the single experiment with 16 biological replicates. RS DI values followed by different letters are significantly different according LSD Fisher test (*P* < 0.05). **(B)** Typical disease symptoms of RS in mock-, BION-, and PSP1-treated plants of RS susceptible variety plants TUCCP 77-42, 9 dpi with a virulent strain of *A. avenae*. Representative leaves are used to illustrate results of each treatment and red stripe symptoms are indicated with arrows.

Induced resistance assays against RS disease in a susceptible sugarcane elite variety was studied under controlled growing conditions spraying whole plants twice with PSP1 (5.1 U/ml) and water (mock) 5 and 2 days before inoculation with a virulent isolate of *A. avenae*. RS severity values of inoculated plants are shown in **Figure 4A**. It is interesting to notice that PSP1 was capable of inducing significant disease protection against RS in sugarcane, although a more prominent protection was seen for plants treated with the commercial product BION (**Figure 4A**). In **Figure 4B** leaves of non-infected and *A. avenae*-infected plants pre-treated with mock (pathogen), PSP1 and BION are shown for a visual demonstration of disease development after the different treatments.

For FHB protection studies in wheat, a small field trial with two varieties ACA 304 (resistant) and DM LYON (susceptible) was conducted during two consecutive growing seasons (2013 and 2014), with a double PSP1-foliar treatment followed by artificial inoculation of the pathogen. As shown in **Table 6**, PSP1-treatment significantly reduced both the incidence and severity of *F*. *graminearum* infection on both genotypes during the two growing seasons studied. Moreover, the induced disease protection was accompanied by a significant increase in TSW. Finally, PSP1-treated plants demonstrated an improved and statistically significant increased GR in *F*. *graminearum*-infected seeds for both genotypes as compared to mock-treated plants in both growing seasons (**Table 6**).

**Table 6:** FHB protection by PSP1 in wheat inoculated with *F. graminearum*.

	Incidence (%)				Severity (%)			
	2013		2014		2013		2014	
Variety	PSP1	Pathogen control	PSP1	Pathogen control	PSP1	CONTROL	PSP1	Pathogen control
ACA 304	6.4 ± 2.1	21.4 ± 3.9a	6.4 ± 1.4a	11.4 ± 2.6a	3.8 ± 1.6a	11.0 ± 3.2a	1.8 ± 0.6a	4.8 ± 1.3a
DM LYON	13.6 ± 2.1a	19.3 ± 2.5a	6.4 ± 2.4a	10.7 ± 2.5a	3.0 ± 0.8a	5.1 ± 0.6a	1.8 ± 0.7a	4.1 ± 1.1a
	10.0 ± 1.7A	20.4 ± 2.3B	6.4 ± 1.3A	11.4 ± 1.8A	3.4 ± 0.9A	8.0 ± 1.8B	1.8 ± 0.4A	4.4 ± 0.8B

	**TSW (g)**				**GR (%)**			
	**2013**		**2014**		**2013**		**2014**	
**Variety**	**PSP1**	**Pathogen control**	**PSP1**	**Pathogen control**	**PSP1**	**Control**	**PSP1**	**Pathogen control**

ACA 304	38.7 ± 0.5a	36.4 ± 0.6a	39.0 ± 0.3a	35.7 ± 0.4b	97.1 ± 0.8a	88.1 ± 1.5a	97.9 ± 0.7a	88.3 ± 2.0b
DM LYON	30.0 ± 1.2a	28.1 ± 1.3a	32.8 ± 1.2b	27.0 ± 0.8c	96.7 ± 1.2a	89.0 ± 2.1a	97.7 ± 0.6a	86.1 ± 1.2b
	34.4 ± 1.4A	32.3 ± 1.3B	36.0 ± 1.0A	31.3 ± 1.3B	96.9 ± 0.7A	88.6 ± 1.3B	97.8 ± 0.4A	87.2 ± 1.2B

*Field trial was performed during two consecutive growing seasons, 2013 and 2014, using the two wheat varieties, ACA 304 and DM LYON. Field trial for each genotype was conducted with a split-plot design with seven blocks where each block was divided in two plots, one corresponding to control- and the other to PSP1-treatment. Seven biological replicates (plots) were assessed for each treatment. Mean and standard error (SE) values of incidence and severity of FHB, thousand seed weight (TSW) and germination rate (GR) are reported for each treatment calculated from seven biological replicates for both years. Different uppercase letters indicate statistically significant differences between PSP1- and mock-treated wheat plants for the different traits considering mean value of both varieties, evaluated according to Tukey’s HSD test (P < 0.05). Different lowercase letters indicate differences between the two genotypes for each variable according to Tukey’s HSD test (P < 0.05).*

## Discussion

In this work, we have described the sequential development of a possible new biostimulant, PSP1, based on an elicitor of natural origin for sustainable disease control in several crop species of importance in Argentina. The technological development of PSP1 followed the steps described for many commercial agricultural products after initial characterization and patenting (Castagnaro et al., 2012), including: (i) the development of a low cost production and downstream-processing system, (ii) formulation for stable storage over longer periods, (iii) toxicology testing on non-target organisms, (iv) a simple handling and application method, (v) criteria of quality control and finally (vi) product registration (Jeyarajan and Nakkeeran, 2000).

Considering that the subtilisin-like protease AsES is the only defense-inducing principle identified until now in the extracellular growth medium of *A. strictum* SS71, fungal growth conditions that stimulated extracellular protein secretion and protease activity were defined as the most important criteria when developing the fermentation process. Previously we have produced AsES by statically growing *A*. *strictum* using PDB as a substrate (Chalfoun et al., 2013) but considering that 30–40% of the production cost of industrial enzymes is estimated to be accounted for by the cost of the growth medium a cheaper alternative (3–5 times), SMB, was tested. Soybean meal is recognized as a potentially useful and cost-effective medium ingredient, because it is largely produced as a by-product during oil extraction (Gattinger et al., 1990) and have been successfully used to promote production of extracellular proteases by filamentous fungi (Agrawal et al., 2005; Haq and Mukhtar, 2007; Savitha et al., 2011). By employing SMB not only did we manage to significantly lower production costs but extracellular protein concentrations increased as well. Furthermore, when scaling up production fermentation times were significantly reduced by more than half demonstrating the usefulness of SMB as substrate for large-scale production of PSP1.

To control residual fungal growth and microbe contamination an acidification step of the supernatant was included in the production process. AsES is an alkaline protease and lowering the pH by acidification of the supernatant to pH 5.5 reduced its activity by 75% (Supplementary Figure 1A) but did not significantly affect its elicitor activity. Thermal variation had very little influence on the protease activity of PSP1 as it retained >90% of its activity when kept at 37°C, 45°C or 60°C at pH 7.5 for 30 min (Supplementary Figure 1B). Interestingly, purified AsES completely lacked enzymatic activity at temperatures above 70°C, whereas PSP1 exhibited some activity even at a temperature as high as 80°C (Supplementary Figure 1C). If this effect is due to enhanced stability of the protein by other constituents in the supernatant or increased instability of the purified protein or a combination of both factors remain to be elucidated. The preservation of enzymatic activity of PSP1 at relatively high temperatures (Supplementary Figure 1C) is in agreement with the observed stability of production batches at temperatures of 37–45°C (**Table 3**).

As previously pointed out we found that a high total extracellular protein concentration of PSP1 was not a perfect indicator of plant disease protection capacity. Therefore, as a previous study had demonstrated that the defense-inducing capability of AsES seems to be directly correlated to the protease activity of this protein (Chalfoun et al., 2013), a measurement of protease activity of PSP1 was tested as a rapid and reproducible quality control method to verify defense-inducing capacity. As all fermentation batches showing detectable protease activity were capable of inducing disease protection and the protease activity has been incorporated as a routine assay of batch quality control. It is however, important to notice that the exact role for this protease activity in plant defense induction is not known and further studies is therefore needed to elucidate the mechanism and possible targets for AsES.

Studies have shown that the concentration of an elicitor can determine the efficacy of the treatment in controlling diseases in different plant species. For example, high concentrations of harpin demonstrated efficient control of *Penicillium expansum* in apple (de Capdeville et al., 2002), whereas lower concentrations were more effective against rice bacterial blight (Chen et al., 2008). In this study we did not find a clear dose–response effect to PSP1-treatment and disease protection in neither strawberry nor soybean. Instead, it was observed that once a minimum protease activity had been reached a full protection was obtained, as increasing the protease activity, protection against strawberry anthracnose or STS did not increase. This threshold effect is in agreement with a previous study showing that the fungal supernatant derived from PDB*-*grown *A. strictum* culture diluted until 1:8 was able to induce full protection (equivalent to higher concentrations) against strawberry anthracnose but this effect was completely lost in a dilution 1:16 (Chalfoun, 2009). Nonetheless, this observation of a lack of dose-effect is somewhat contrasting to results obtained in Arabidopsis where an AsES dose–response was indeed observed in disease protection against the necrotrophic fungus *Botrytis cinerea* (Hael-Conrad et al., 2015). These differences could be explained by different challenging pathogens-plant systems, and differences between effects of PSP1 and purified AsES.

The *sine qua non* mandatory requirement for the registration of a biological product is the complete lack of animal toxicity and negative effects on the environment as they are intended to be used as tools in sustainable crop production and/or important components in IPM protocols or organic farming. Thus, the toxicity effect of PSP1 on non-target organisms such as insects, fish, mammals and birds was tested following IOBC standards for extended laboratory tests on natural substrates in accordance with guidelines of the National Food Safety and Quality Service (SENASA) for registration of a microbial biological control agent (ACBM), a microbial technical product (PTM) and a formulated microbial product (PMF) in Argentina. It is important to notice that there are no guidelines or even a category for registration in Argentina of compounds of natural origin to be used in agriculture practice, such as biostimulants. This fact has been pointed out to responsible authorities during the ongoing registration process of PSP1 and work has been initiated to include such products in the registration procedure in the future. Most importantly though, as demonstrated by these studies no adverse effects of this product are expected in field applications on the environment, animals or field workers and the PSP1 formulation should be a perfect complement in any type of sustainable crop disease management by itself or in combination with other products.

The development of biocontrol products based on plant defense elicitors such as MAMPs/PAMPs/DAMPs is a promising strategy for sustainable crop disease management, because they can render protection against a broad spectrum of plant pathogens in a wide-range of different plant species. However, elicitor molecules are specifically recognized in different host species and therefore induce different defense responses and so different protection level against diseases (Reboutier et al., 2007). For this reason it is important to thoroughly test defense induction and to evaluate potential disease control capacity in different crop species.

Soybean is by far the most important crop in Argentina and it was a logic step to test for disease protection capability of PSP1 in this species. The necrotrophic late season disease pathogen *C. cassiicola*, causing STS, which completely destroys the leaf cell as a strategy to obtain nutrients (Onesirosan et al., 1975; de Lamotte et al., 2007), was chosen for the initial IR studies. The occurrence of target spot on soybean has caused significant yield losses worldwide (Yorinori, 1997; Sinclair, 1999) and in recent years, due to the occurrence of *C. cassiicola* isolates resistant to fungicides, epidemics of the disease have been frequent in many soybean-growing regions in Brazil (Godoy et al., 2012; Teramoto et al., 2013) and Argentina (Ploper, 2011). No commercially relevant resistant cultivars are currently available and seed treatment or foliar spray with fungicides together with crop rotation are currently the most used disease control strategies (Almeida et al., 2005).

The time of application of an elicitor is a critical parameter in regard to its efficacy in controlling a plant disease. Generally elicitors/PAMPs are applied 2–5 days prior to a pathogen invasion (Qiu et al., 2001; de Capdeville et al., 2002, 2003). For example Messenger^®^ formulated with the bacterial protein harpin was effective to control blue mold disease in apple when applied 2 days before pathogen inoculation (de Capdeville et al., 2002) and the scab disease in rough lemon when applied 5 days before infection. However, the same product failed to control the disease when applied 10 days previous to pathogen inoculation (Agostini et al., 2003). In strawberry optimal anthracnose protection was obtained in plants pretreated with a supernatant of *A*. *strictum* 7 days before pathogen inoculation although good protection was also observed in plants treated 3 days previous to infection (Chalfoun, 2009). In accordance with these latter results PSP1-treatment of soybean plants was performed 3 and 7 days before *C*. *cassiicola* infection together with a 3 days post infection treatment. PSP1-treatment was effective against STS when applied 3 days before pathogen infection but this effect was markedly reduced when the time between PSP1-treatment and pathogen challenge increased to 7 days. Somewhat surprisingly a good protection was observed for plants treated with PSP1 3 days after infection with *C. cassiicola* although PSP1 do not possess any fungicide activity, indicating that PSP1-treatments 3 days before until 3 days after infection are effective against target spot and that the PSP1-induced response of the plant was able to combat the fungus in early stages of infection.

For soybean there are several reports demonstrating control of fungal diseases, although none of them have shown protection against *C*. *cassiicola*, by application of elicitor-based products under controlled growing conditions (Dann et al., 1998; Prapagdee et al., 2007; Nafie and Mazen, 2008; Lemes et al., 2011; Han et al., 2013; Cruz et al., 2014). However, there are only three cases in soybean, where elicitor-treatment have been described to be effective against necrotrophic pathogens; i.e., soil applications of mineral nutrient silicon (Si) reducing the incidence of frog-eye leaf spot and downy mildew diseases (Nolla et al., 2006), Regalia^®^-treatment controlling Cercospora leaf blight caused by *Cercospora kikuchii* (Su et al., 2011), and EplT4, a peptide from *Trichoderma asperellum* T4, applied 12 h before infection protecting plants against *Cercosporidium sofinum* (Wang et al., 2013).

Once application dosage and treatment times for PSP1 had been optimized for target spot protection in soybean, a comparison of disease protection efficacy with the commercial elicitor BION was conducted. The active compound in BION is the SA-analog, BTH, which has been shown to be effective against a wide range of soybean pathogens causing damaging soil-borne diseases (Dann et al., 1998; Faessel et al., 2008; Nafie and Mazen, 2008; Abdel-Monaim et al., 2012; Sugano et al., 2012; Han et al., 2013). As demonstrated by our results plants treated with BION or PSP1 showed a similar protective effect against *C*. *cassiicola* in soybean and a very similar plant defense response as shown by accumulation of ${\text{O}}_{2}^{•-}$ and H_2_O_2_ 4 h post-treatment and a strong induction of *GmPR1* expression. The latter result demonstrated that both elicitors were able to activate the SA-mediated signaling pathway in this species, although a noticeable difference in *GmPR1* induction kinetics was observed with an earlier and stronger gene induction in PSP1-treated plants as compared to BTH-treated ones (Lawton et al., 1996). This variation is probably dependent on the difference in SA-signaling, as previous studies have shown that treatment with AsES induces a transient accumulation of SA in strawberry (Chalfoun et al., 2013; Hael-Conrad et al., 2018) and Arabidopsis (Hael-Conrad et al., 2015), whereas BTH is acting downstream of SA accumulation (Friedrich et al., 1996). However, more studies on hormonal signaling pathways induced by AsES/PSP1 are needed to establish if there are any differences in the underlying molecular mechanisms of pathogen protection among different plant species.

The encouraging results from PSP1 application in soybean prompted us to test protection against diseases in an important crop species in the Northwest of Argentina. The reason behind selecting sugarcane and red stripe was twofold, first we wanted to test PSP1 in a monocot species and secondly to test if protection could be achieved against a bacterial disease as only fungal diseases had been tested previously. Although symptoms of red stripe in sugarcane were reported as early as 1895 in Argentina, the disease has not been considered a major problem until the last decade. The vastly increased number of incidents of this disease has been accompanying the implementation of new agricultural techniques in Argentina such as green-cane harvesting and crop rotation with soybean (Fontana et al., 2013). This situation has caused an increasing attention to the disease and because red stripe can significantly decrease sucrose recovery in susceptible varieties there is great concern how to reduce infection rates (Johnson et al., 2016). Actually, the only effective way for controlling red stripe in sugarcane has been the replacement of susceptible commercial varieties with more resistant ones (Martin et al., 1989) and it is therefore of great interest to develop alternative disease management strategies to be able to better control the disease in susceptible sugarcane varieties with good agronomical characteristics. The induced disease protection against red stripe in a susceptible sugarcane variety indicated that application of PSP1 is not only an interesting alternative for red stripe control in sugarcane but also suggested that PSP1 is a viable alternative for disease control in monocots. In addition, this is the first example of a successful protection against a bacterial disease mediated by AsES. These results taken together strongly indicates that AsES is acting as a PAMP/DAMP inducer and that the signaling is well conserved within the plant kingdom as has been seen for other proteinaceous inducers originating from microorganisms such as harpin and flagellin (or the peptide Flg22).

In parallel with testing PSP1 in sugarcane a 2-year field trial in wheat was conducted to test protection against *F.* *graminearum*, the principal causal agent of FHB in cereals. FHB is a devastating disease causing important losses in the commercial value of grains in both wheat and barley by reducing grain yields and by production of mycotoxins harmful to both humans and animals (Goswami and Kistler, 2004). Wheat is the most important winter cereal in Argentina with more than 5.000.000 ha planted and several FHB outbreaks have occurred during the last 60 years with estimated yield losses between 20 and 50% (Stenglein, 2009). The two main strategies to control this disease have been development of resistant plant varieties and application of broad range fungicides, but neither of these approaches have been very successful (Mesterhazy, 2003; Trail, 2009). Due to the lack of efficient disease management of this important disease, several studies on alternative biological treatments have been tested. An isolate of *Pseudomonas fluorescens* was reported to enhance resistance to Fusarium seedling blight and head blight caused by *Fusarium culmorum* in wheat by inducing both local and systemic responses (Khan et al., 2006; Khan and Doohan, 2009a,b). Further studies revealed that plant hormones indole acetic acid (IAA) and abscisic acid are involved in the *P. fluorescens*-mediated control of FHB in barley and that application of IAA to plants grown at mid-anthesis 24 h before pathogen infection, reduced FBH symptoms and increased yield (Petti et al., 2012). From these results it was suggested that IAA might offer a realistic treatment for the control of diseases such as FHB, where crops have a limited and clearly defined period of infection, mid-anthesis in the case of FHB (Petti et al., 2012). A significant protective effect against wheat FHB caused by *F. graminearum* has also been demonstrated for the known defense-inducer BABA (Zhang et al., 2007). Surprisingly, foliar application of BTH has widely been reported to reduce the severity of various fungal infections in wheat but it does not provide resistance to FHB (Yu and Muehlbauer, 2001). In addition to the above cited studies on successful application of compounds of natural origin for FHB management, results from the 2-year field trial studying diminished seed weight and viability caused by *F. graminearum* indicated that PSP1 provides an additional tool to effectively control this pathogen. The weight value of a thousand seeds constitutes a good parameter to measure yield loss caused by the inoculation with *Fusarium* spp. causing FHB and has previously been used by other authors (Petti et al., 2012). However, to confirm that this observed effect is reproducibly significant at total yield level, it is necessary to perform field trials determining total yields of plots under different growth conditions.

In this later study, we demonstrated that disease protection against *F. graminearum* by application of PSP1 was dependent on the plant genotype, since a greater impact was observed for the more susceptible genotype. This observation has previously been reported for other pathogen defense-inducing compounds including INA and BTH, which both showed better protection on more susceptible cultivars of soybean when challenged with *Sclerotinia sclerotiorum* (Pawlowski, 2015). The application of INA to soybean reduced natural infection of white mould by 20–70% in cultivars that are considered highly susceptible to this fungus, whereas the effect was much lower in resistant cultivars (Dann et al., 1998). However, effects of treatments in wheat has been more variable as application of JA resulted in an 85% decrease in *Tilletia indica* infection levels in susceptible varieties compared to 50% in resistant ones (Dutt et al., 2011), whereas Desmond et al. (2006) showed that MeJA treatment significantly delayed development of crown rot disease caused in wheat seedlings by the necrotrophic fungus *Fusarium pseudograminearum* in a genotype-independent manner.

From the combined disease protection studies in strawberry, soybean, sugarcane, and wheat it is obvious that although PSP1 is capable of inducing the plant defense against pathogens in all four species, it is necessary to optimize application procedure of PSP1 for maximum disease protection in the individual plant species.

It is important to observe that for all disease protection assays reported in this study, pathogens were artificially infested, and that all experiments except the wheat trial were performed under controlled growing conditions. This situation is very different from field growing conditions where plants are continuously exposed to pathogens and climate changes, constantly activating different biotic and abiotic defense responses as well as affecting plant hormones homeostasis. Another important factor to take into account is the possible energy costs generated by defense-inducing treatments against pathogens in the field, especially under poor nutrition conditions. For example, fitness costs of BTH-induced resistance in wheat were observed when nitrogen was limiting but undetectable in well fertilized plants (Heil et al., 2000). Thus the ability or requirement to respond to a pathogen attack may be reduced or unnecessary, but will be energy requiring and could therefore negatively affect yields, especially under poor nutrition conditions (Smedegaard-Petersen and Tolstrup, 1985).

It is therefore important to conduct multi-fold field trials under different climate conditions to be able to properly evaluate the protective effect of PSP1, to get an idea of their true value in crop disease management as there are several examples of defense activators that have performed well when used to control pathogen infections in laboratory or greenhouse conditions but have shown greater variability when applied in field environment (Hopkins, 2002; Agostini et al., 2003; Graham and Leite, 2004; Obradovic et al., 2005; Keinath et al., 2007; Walters and Fountaine, 2009). This inconsistency has in many cases hindered their establishment as proper components of disease control programs in crop production.

In summary, it is highly encouraging that a good disease protection was obtained in different crop species and toward different pathogens by PSP1-treatment and that in at least one case the protection was observed under natural growth conditions. These results show that PSP1 could be a valuable and sustainable alternative to chemical pesticides for successful control of a large number of diseases in important crop species, although additional field trials are required before a definitive answer to this postulation can be given.

## Author Contributions

NC, SD, EM, AC, and BW conceived and designed the experiments. NC performed the experiments in strawberry and soybean. SD carried out the optimization of the fermentation process and its scaling-up. NC and SD analyzed and interpreted the data. FB and MC carried out the analytical measurements of protein. PDP optimized the phytopathological test in soybean. RB performed the phytopathological assays in sugarcane. SS carried out the wheat field trials. NC and BW drafted the work and wrote the manuscript. AC revised critically the article. NC edited the manuscript. All authors approved the final version of the manuscript.

## Conflict of Interest Statement

The authors declare that the research was conducted in the absence of any commercial or financial relationships that could be construed as a potential conflict of interest.
