# Supplementary material for: Development of PSP1, a Biostimulant Based on the Elicitor AsES for Disease Management in Monocot and Dicot Crops
Source: Front Plant Sci. 2018 Jul 24;9:844. doi: 10.3389/fpls.2018.00844 (PMC6066549; doi:10.3389/fpls.2018.00844)

**Supplementary Figure 1. Enzymatic properties and thermal stability of purified AsES and PSP1.** The proteolytic activity of AsES was characterized using the chromogenic peptide Suc-AAPF-*p*NA as a substrate. (A) Purified AsES (0.82 µg/ml) and PSP1 (0.74 µg TSP/ml) were assayed at the indicated pH for 30 min at 37 ºC. Buffers used in the assay were 200 mM sodium acetate (pH 4.0 and 5.0), 200 mM sodium phosphate (pH 6.0, 7.0, 7.5 and 8.0), and 200 mM glycine–NaOH (pH 9.0 and 10.0). (B) The same samples used in A were assayed at 37, 45, 60, 70 and 80^o^C for 30 min in 20 mM Tris–HCl, pH 7.5. (C) The same samples used in A were assayed at 37°C for 30 min in 20 mM Tris–HCl, pH 7.5 after incubation at the indicated temperatures for 30 min to measure residual protease activity. Each value represents the mean ± SD from triplicate determinations. Specific activities of pure AsES and the PSP1 at 37ºC and pH 7.5 are 2.8 mmol pNA min^-1^ mg AsES^-1^ and 2.01 mmol pNA min^-1^ mg TSP^-1^, respectively.


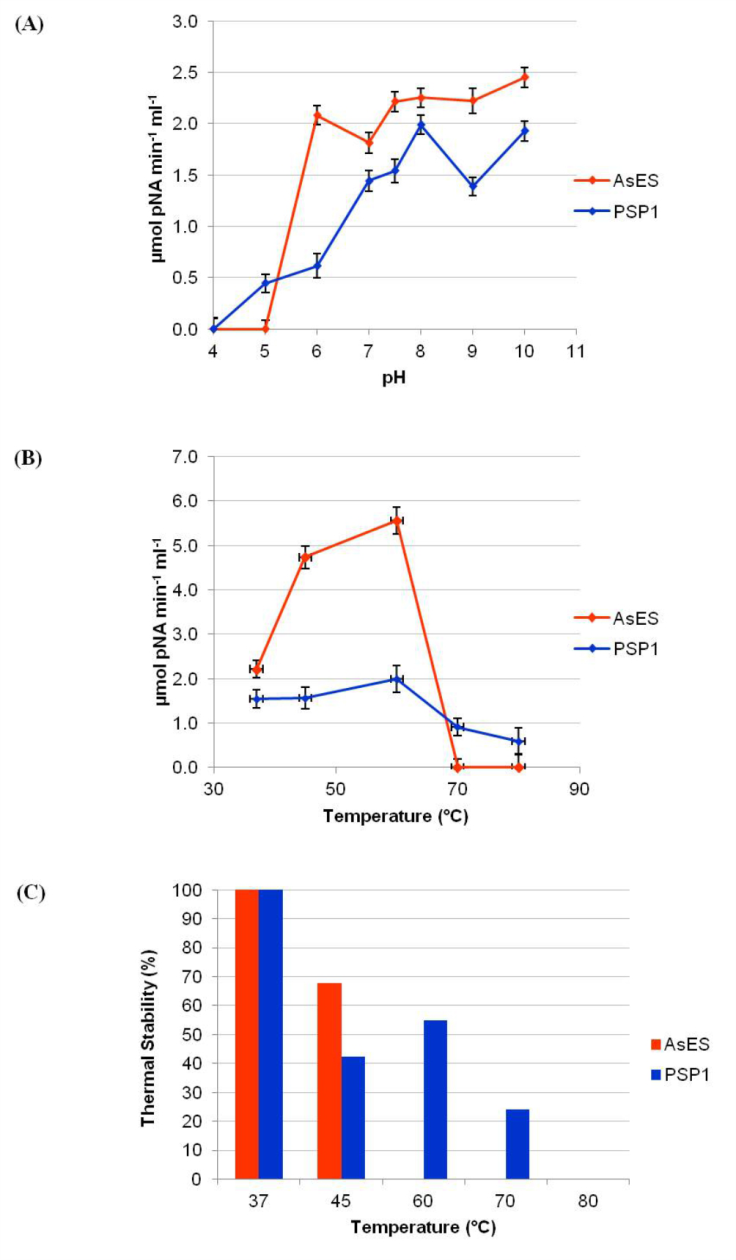

Supplement: Supplementary file 1 [file Data_Sheet_1.DOCX]
